# Current Role of the Nonsteroid Treatment of Idiopathic Sudden Sensorineural Hearing Loss (ISSNHL): A Narrative Review

**DOI:** 10.3390/jcm14082811

**Published:** 2025-04-18

**Authors:** Concepción Rodríguez-Izquierdo, Mayte Herrera, Anastasiya Avdiyuk, Daniel Rodríguez-Ocaña, Guillermo Plaza

**Affiliations:** 1 Otorhinolaryngology Department, Hospital Universitario de Fuenlabrada, 28042 Madrid, Spain; mariateresa.herrera@salud.madrid.org (M.H.); anastasiyaavdiyuk43@hotmail.com (A.A.); drodocana@gmail.com (D.R.-O.); guillermo.plaza@salud.madrid.org (G.P.); 2 Otorhinolaryngology Department, Hospital Universitario Sanitas La Zarzuela, 28023 Madrid, Spain; 3 Departamento de Especialidades Médicas y Salud Pública, Facultad de Ciencias de la Salud, Universidad Rey Juan Carlos, 28933 Madrid, Spain

**Keywords:** sudden sensorineural hearing loss, treatment, hyperbaric oxygen therapy, exploratory tympanotomy, nonsteroid treatment, prostaglandin

## Abstract

Sudden sensorineural hearing loss (SSHNL) is an abruptly appearing hearing loss. The etiology remains unclear, although vascular pathologies, viral infections, or autoimmune disease contribute to the understanding of this pathology. Systematic steroids are often used as the first-line treatment because of their anti-inflammatory effect. However, there remains controversy about the use of steroids and other alternative treatments, as hyperbaric oxygen therapy (HBOT), exploratory tympanotomy, prostaglandin, N-acetylcysteine, or defibrinogenation therapy. In this study, we aim to review the various treatment options currently available for sudden hearing loss, with the objective of advancing our understanding of this condition and clarifying information to guide future clinical practice guidelines.

## 1. Introduction

Sudden sensorineural hearing loss (SSNHL) is one of the most significant otologic emergencies. It is defined by most authors as the onset of sensorineural hearing loss of 30 dB or greater in three or more consecutive frequencies on tonal audiometry that develops within three days or less [1]. The incidence varies significantly across different countries, generally being less than 100 cases per 100,000 people per year, primarily affecting adults between the ages of 45 and 55 [2]. However, since the estimated spontaneous recovery rate is between 32% and 65%, it is believed that its actual incidence may be higher [3].

The etiology of SSNHL can be multifactorial: vascular, autoimmune, viral, or traumatic (membrane rupture). However, the cause can only be determined in 10–15% of cases [1,2,3,4]. Cases where the etiology cannot be identified are considered idiopathic (ISSNHL), making it a diagnosis of exclusion.

There is no approved medical treatment for ISSNHL. Although the administration of steroids, both locally and systemically, is widely accepted for reducing inflammation and the immune response in the cochlea and auditory nerve [3], there is still no clear evidence of their efficacy.

**Figure 1:**
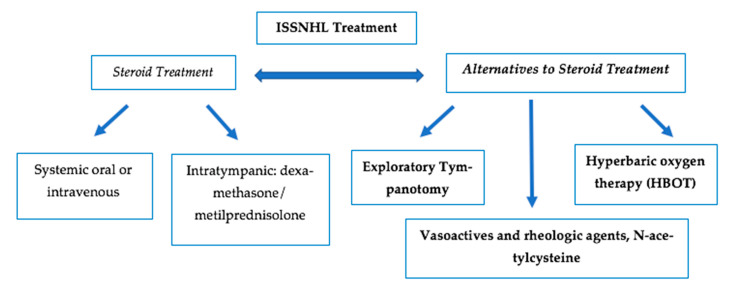
Multimodal treatment options for ISSNHL.

This study aims to review the main treatment options alternative to corticosteroids (exploratory tympanotomy, hyperbaric oxygen therapy, and others, like prostaglandins or N-acetylcysteine) in ISSNHL (Figure 1). Due to the vast amount of information available, articles that synthesize information are necessary to aid in the therapeutic management of ISSNHL and the creation of clinical guidelines.

## 2. Methodology

For the completion of this work, the authors conducted a rigorous review of the literature published from October 1979 to January 2025. The databases used were PubMed/MEDLINE, EMBASE, and the Cochrane Library, employing the following terms: hearing loss, sudden; sudden hearing loss; sudden deafness; sudden sensorineural hearing loss treatment; idiopathic sudden hearing loss; hyperbaric oxygen therapy; and exploratory tympanotomy.

**Figure 2:**
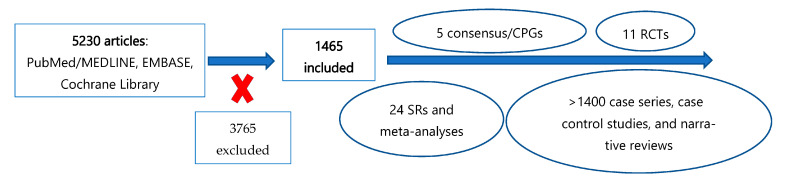
Methodology followed in this narrative review. CPGs: Clinical Practice Guidelines; RCT: randomized controlled trial; SR: systematic review.

A total of 5230 articles in Spanish and English were retrieved, with 3765 being excluded for lack of relevance (Figure 2). Special attention was given to systematic reviews, meta-analyses, randomized clinical trials, clinical practice guidelines, and consensus statements. The quality criteria we used to identify this series of articles are described in Table 1. However, other types of articles (case series, case–control studies, and narrative reviews) were also included. Given the significant heterogeneity in the treatments for sudden hearing loss and the primary objective of this study being a review of non-steroidal treatment options, it was necessary to expand the scope by incorporating information from these types of studies.

## 3. Development of Steroid Treatment

Among the different therapeutic options, corticosteroids have been the main medical treatment in ISSNHL [1,2,3,4,5] since the publication of the Wilson et al. study in 1980 [6]. The use of corticosteroid therapy was especially reinforced with the acceptance of immune-mediated inner ear disease as a clinical entity due to its possible relationship with the autoimmune etiology of sudden hearing loss [7].

**Figure 3:**
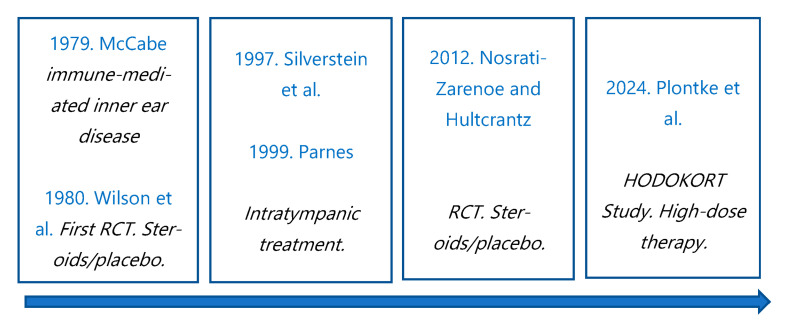
Corticosteroid treatment for ISSNHL over the years [6,7,10,11,12,13].

Throughout history, steroid treatment has been investigated and reviewed in its different routes (Figure 3). In general, the most common initial route of administration is systemic, with oral corticosteroids in a tapering dose being the most widely accepted treatment [8,9]. The intratympanic and intravenous routes are reserved for more specific cases, such as rescue therapy and severe cases, respectively. The steroids used for the treatment of sudden hearing loss include prednisone (60 mg), methylprednisolone (48 mg), and dexamethasone (10 mg) [3].

In 1997, Silverstein et al. [10] first described the intratympanic route as an alternative to systemic treatment, with Parnes’ experimental work in 1999 [11] being fundamental in understanding the pharmacokinetics of steroids in the inner ear. The advantages of the intratympanic route are numerous [14], as it is an outpatient procedure that is generally well tolerated. Due to the semipermeable properties of the round window, it allows medications administered intratympanically to pass through the hematocochlear barrier via pinocytosis and diffusion [15]. This results in a higher concentration of the drug in the perilymph compared to systemic administration [9,16,17]. Additionally, systemic absorption is minimal, reducing the risk of side effects.

The publication of these studies was subsequently supported by case series and retrospective studies [18,19], generally endorsing the empirical use of steroids.

Systematic reviews and meta-analyses [20,21] were later conducted, thoroughly examining the published works, and starting to question the systematic use of corticosteroids in ISSNHL, mainly due to the significant limitations of the supporting studies (small sample sizes, variability in the definition of ISSNHL, and differing treatment regimens). With the publication of the triple-blind randomized clinical trial by Nosrati-Zarenoe [12] in 2012, which included 93 patients treated with prednisone or a placebo, it was concluded that oral corticosteroids at standard doses did not improve hearing in patients with ISSNHL. These findings were reinforced by further reviews and meta-analyses, casting doubt on the effectiveness of both systemic [22,23] and intratympanic corticosteroid treatment.

The use of intratympanic corticosteroids (ITCs) is of particular interest due to their importance in the treatment of sudden sensorineural hearing loss (SSNHL). Throughout history, there has been significant heterogeneity in the publication and results of studies on intratympanic corticosteroids, varying in type (dexamethasone/methylprednisolone), timing of infiltration (initial or salvage treatment), combination with systemic corticosteroids (sequential or simultaneous), and infiltration protocols (daily, weekly, number of doses).

Initially, it seemed that combined therapy (systemic and intratympanic corticosteroids) could be beneficial. Battaglia et al. [24], in their study on combined therapy with oral and intratympanic corticosteroids, and Han et al., in their systematic review published in 2017 [25], suggested that this therapy could lead to improvements in hearing. However, when the treatment protocols of these studies were later analyzed in a meta-analysis using a mathematical model, it was not possible to conclude that the final improvement in hearing depended on the type of treatment, frequency, duration, or dosage but rather on the severity of the hearing loss [26].

In July 2022, Plontke et al. [27] published a Cochrane review critically assessing the effectiveness of intratympanic corticosteroids due to their limited demonstrated effect (around 10 dB) and the low certainty derived from the poor quality of the trials. The authors analyzed intratympanic corticosteroids as a salvage treatment (showing an effect five times more favorable for ITC rescue compared to a placebo), ITCs versus systemic corticosteroids as a primary standalone treatment, and ITCs versus ITCs combined with systemic therapy.

Regarding salvage treatment with intratympanic corticosteroids, a study published in 2020 by Andrianakis et al. [28] compared two infiltration protocols: one group received three infiltrations at weekly intervals, while the other followed a shorter interval between doses (2–4 days) with a higher number of infiltrations (four), as suggested by the AAO-HNSF in 2019 [2]. The authors found no significant differences between the two treatment groups. However, they suggested that adding a fourth dose did not appear to have a significant effect, aligning with the conclusions of other authors like Suzuki et al. [29]. In a recent systematic review, Li et al. [30] analyzed a total of 16 studies, concluding that most authors generally agree on the efficacy of this treatment modality. However, they emphasized that the major limitation of these studies was their heterogeneity and lack of methodological rigor. Another example of the wide variety of intratympanic treatment is the retrospective study published by Andrianakis, A et al. [31], in which they reviewed patients treated with triamcinolone acetonide as a rescue treatment after systemic corticosteroids. They found significant improvements in hearing, with results similar to those of dexamethasone and methylprednisolone.

The discrepancy in the results regarding the use of corticosteroids in the treatment of ISSNHL persists to this day. For example, the recent publication by Plontke et al. [13], based on the HODOKORT study, analyzed a total of 325 patients with moderate ISSNHL who were randomized to receive high-dose oral corticosteroid treatment (dexamethasone) or intravenous (prednisone) corticosteroids, with a descending-dose oral corticosteroid group (prednisone) serving as the control group. The authors found no evidence supporting the efficacy of high-dose corticosteroid treatment, though the associated side effects were significant.

On the other hand, a new intratympanic therapy recently introduced by Rommelspacher et al. [32] is the infiltration of 6-fluoro-9-methyl-pyridoindole (AC102), which has shown promising results in an animal model (guinea pig). This treatment demonstrated hearing recovery in cases of hearing loss related to noise exposure. The study revealed that this molecule protected against cellular apoptosis, reduced reactive oxygen species (ROS) levels, and enhanced neuronal growth—key aspects in the pathophysiology and treatment of sudden hearing loss. In fact, this molecule is currently being evaluated in a phase II study by comparing its effectiveness with oral corticosteroids as a treatment for ISSNHL.

Therefore, exploring other treatment options seems reasonable to achieve better outcomes in the treatment of ISSNHL.

## 4. Alternatives to Steroids in ISSNHL

### 4.1. Exploratory Tympanotomy

The etiology of ISSNHL is known to be multifactorial. The membrane disruption theory as a cause of ISSNHL was proposed over 50 years ago. In 1970, Stroud and Calcaterra [33] described the first case of spontaneous perilymphatic fistula, followed by Goodhill, who identified the main mechanisms of origin as well as perilymphatic fistula as a cause of sudden hearing loss [34]. Thus, the rupture of the oval or round window membrane can be considered a possible etiology of this condition, with potential causes including middle ear malformations (e.g., abnormal stapes superstructure), inner ear malformations (e.g., enlarged vestibular aqueduct or Mondini malformation), iatrogenic causes and barotrauma, or even spontaneous rupture [35].

The role of exploratory tympanotomy (middle ear surgical exploration) in SSNHL treatment has been debated for years, with no consensus on its routine use. Currently, some authors suggest considering this technique in cases of severe-to-profound idiopathic hearing loss that fail to improve after medical treatment. This applies even in the absence of classic symptoms suggestive of perilymphatic fistula, such as vertigo, tinnitus, or barotrauma history [35,36,37].

The literature describes three main criteria for confirming the presence of a perilymphatic fistula during exploratory tympanotomy: direct observation of perilymph leakage during the Valsalva maneuver, visualization of a rupture in the oval or round window membrane, or demonstration by increasing the intracochlear pressure through gentle palpation of the stapes. Other less common diagnostic methods include the detection of beta-trace protein, cochlin tomoprotein (CTP), or intraoperative visualization of flow following the administration of intrathecal fluorescein [38,39,40,41]. Unfortunately, there is no pathognomonic finding that can standardize the diagnosis of perilymphatic fistula.

In fact, in many cases described as successfully treated with fistula sealing, none of the three criteria were confirmed during surgery [42]. In contrast, other authors report the presence of fluid in the round window in 40–71% of cases, particularly in those explored during the first week after symptom onset [43]. Despite ongoing controversy, there is increasing interest in using exploratory tympanotomy with round window sealing, regardless of whether a perilymphatic fistula is observed [35]. This procedure is considered a rescue treatment when other approaches fail.

Some authors suggest that the favorable outcomes of tympanotomy with round window sealing in patients with SSNHL—without evident perilymphatic fistula during surgery—might be due to improved perfusion or blood flow induced by the surgical procedure rather than the sealing itself and the presence of micro-ruptures, spontaneous recovery, or a fluctuating fistula that resolves when the patient is in a supine position [35].

Typically, this treatment is reserved for patients with severe-to-profound hearing loss, preferably in individuals under 65, and often associated with vertigo [42]. It is recommended to perform the procedure within 5–10 days of symptom onset [35,37,44,45].

In their review of 136 ISSNHL cases treated with exploratory tympanotomy, Thomas et al. [37] reported a 77% improvement in hearing, with the improvement being particularly significant in patients who had a history of pressure changes. On the other hand, Heilen et al. [44], after reviewing 300 cases included in the literature along with 9 of their own, did not find a clear improvement in hearing regardless of the presence of a fistula. Additionally, tympanotomies showed approximately 13.6% of fistulas.

**Figure 4:**
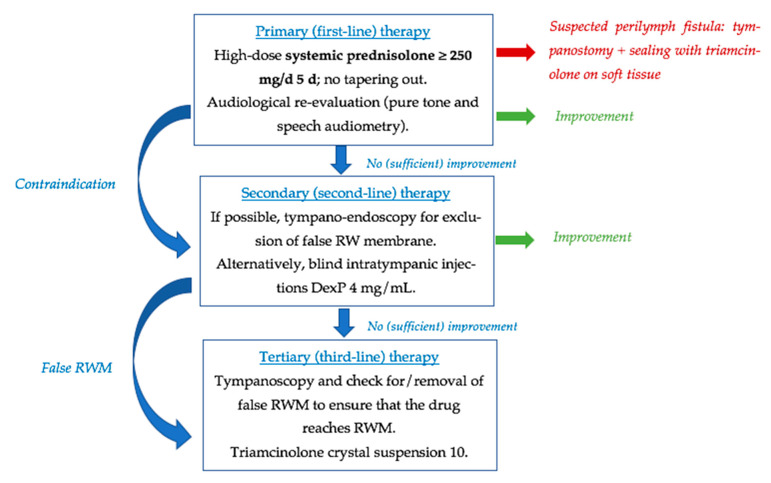
Stage approach to therapy of sudden hearing loss. Published by Plontke et al. [7]; used under the Creative Commons Attribution 4.0 (CC BY 4.0) license.

According to the current clinical guidelines in Germany [7], exploratory tympanotomy is recommended for patients with persistent severe hearing loss (>80 dB) after five days of intravenous prednisolone treatment at 250 mg/day [Figure 4]. Nevertheless, neither the current consensus in the United States nor in Spain recommends exploratory tympanotomy as a treatment for ISSNHL [1,2,40].

### 4.2. Hyperbaric Oxygen Therapy (HBOT)

Hyperbaric oxygen therapy (HBOT) consists of breathing pure oxygen (100%) at increased pressure in a hyperbaric or pressurized chamber. This leads to an elevation in partial pressure of oxygen (PaO_2_) and plasma oxygen levels, facilitating enhanced cellular exchange through capillaries, even in areas compromised by ischemic hypoxia.

Decreased oxygenation in cochlear tissues is a potential etiology of ISSNHL. The inner ear receives its main blood supply from the labyrinthine artery, which has three branches: the vestibulocochlear artery (VCA), the anterior vestibular artery (AVA), and the main cochlear artery (MCA). One of the specific possible causes of sudden hearing loss is the occlusion of the VCA, a syndrome described by Murofushi and colleagues called “vestibulocochlear artery syndrome” [46]. This syndrome is generally associated with a loss of function in the posterior semicircular canal while preserving saccular function. It is typically accompanied by vertigo and hearing loss at high frequencies. VCA occlusion can have multiple causes. Several meta-analyses and systematic reviews have shown that elevated total cholesterol levels are associated with a higher risk of ISSNHL [47,48,49]. The correlation between diabetes mellitus, hypertension, and a history of myocardial infarction with ISSNHL risk has also been demonstrated, though the results are less conclusive [47]. In a study by Park et al. [50], patients with ISSNHL were 1.69 times more likely to develop cardiocerebrovascular disease within the first 12 months of follow-up compared to matched controls (HR = 1.69, CI = 1.46–1.94).

Given the possible vascular–ischemic etiology, HBOT has gained importance as a treatment for ISSNHL by increasing the oxygen concentration in the inner ear [48]. The updated Clinical Practice Guideline of the American Academy of Otolaryngology–Head and Neck Surgery (AAO-HNS) suggests HBOT as an optional initial treatment (combined with corticosteroids) within the first two weeks of symptom onset or as a rescue therapy within one month [2]. Similarly, the Japanese Clinical Practice Guideline for sudden hearing loss [51] recommends HBOT within two weeks of symptom onset (Level of Evidence I, Recommendation Grade C1) but advises against its use once symptoms stabilize.

Numerous studies, including prospective studies, systematic reviews, and meta-analyses, have demonstrated the superiority of HBOT combined with corticosteroid therapy compared to corticosteroid therapy alone [52,53,54,55,56,57,58,59,60,61,62,63,64]. Bayoumy et al. [54] emphasized the importance of early HBOT initiation, stating that timing is the most critical factor for its effectiveness. They recommended considering ISSNHL as an otologic emergency. Rhee et al. [55] analyzed 2401 patients from 19 studies: 3 randomized controlled trials, 2 prospective studies, and 14 retrospective observational studies. Their study concluded that patients who received HBOT along with medical therapy experienced either complete hearing recovery or some degree of improvement: OR = 1.61, 95% CI = 1.05 to 2.44; OR = 1.43, 95% CI = 1.20 to 1.67, respectively. The benefits were more pronounced in patients with severe-to-profound ISSNHL (≥70 dB) when HBOT was used as salvage therapy or applied for more than 1200 min.

Although Rhee et al. initially faced criticism for heterogeneity in their review, subsequent studies with stricter inclusion criteria have yielded similar results. Joshua et al. [53] included 150 patients with ISSNHL (three prospective randomized controlled trials). When used as an adjunctive therapy, HBOT significantly improved absolute hearing gain (MD = 10.3 dB, 95% CI = 6.5 to 14.1, I^2^ = 0%) and hearing recovery (OR = 4.3, 95% CI = 1.6 to 11.7, I^2^ = 0%) compared to medical therapy alone. They recommended applying HBOT for at least 900 min at 2.0 atmospheres absolute (ATA) for patients experiencing ISSNHL at any level of severity. Moody-Antonio et al. [56] highlighted the value of HBOT combined with corticosteroids, especially in patients with severe-to-profound hearing loss.

In 2023, Alter et al. [57] reviewed multiple systematic reviews and meta-analyses (Table 2). They concluded that although some studies report mixed results, the overall evidence suggests that HBOT may be effective in treating ISSNHL, particularly when started early and used as an adjunct to steroid therapy. These results were reinforced by other recent systematic reviews, such as those by Liu X et al. [58] and Sánchez-Lozano [59]. Liu X et al. reviewed nine systematic reviews and meta-analyses; six reported HBO’s effectiveness, while three found no significant difference between HBO and alternative therapies. Meanwhile, Sánchez-Lozano’s review included a total of eight randomized clinical trials with 806 participants, highlighting the great heterogeneity among HBOT treatment protocols as the main limitation.

In regard to comparing the efficacy of HBOT and intratympanic corticosteroids (ITCs) as salvage treatments following the failure of systemic corticosteroid therapy, Kuo et al. [64] recently conducted a meta-analysis including three observational studies and one randomized controlled trial. No statistically significant differences were observed in hearing gain between the two treatments. The authors conclude that HBOT and ITCs might exert a synergistic effect when combined, as they operate through different mechanisms: HBOT increases oxygen availability and ITCs have a strong anti-inflammatory effect. The authors highlighted that spontaneous recovery in ISSNHL can range from 32% to 65%, complicating the interpretation of treatment efficacy. They recommended selecting a treatment based on the experience of the medical team and the availability of resources, as both approaches yield similar outcomes with minimal adverse effects. Yang et al. [65] conducted a cohort study comparing the treatment of ISSNHL with ITCs alone, HBOT alone, or a combination of both. They found that combination therapy may improve word recognition scores, as well as result in greater recovery of hearing, especially at lower frequencies.

Lei et al. [61] compared HBOT and ITCs for salvage therapy in another meta-analysis. A trend suggested that ITCs might be superior to HBOT, but no statistically significant differences were observed between the two treatment modalities. The review included six studies, most of which had similar characteristics except for the one by Sun et al. [66], which focused on refractory high-frequency ISSNHL. The limitations of the meta-analysis were small sample sizes in the studies reviewed and few randomized controlled trials, partly due to HBOT’s higher cost and longer treatment durations compared to ITCs. Similarly, Lin et al. [67] showed that HBO therapy has no significant additional benefits compared to postauricular steroid injections and ITCs.

On the other hand, Sanda et al. [68] recently published a case–control study evaluating the effectiveness of HBOT in combination with systemic corticosteroids, both oral and intravenous. Their study included 67 patients in an HBOT group and 68 patients in a non-HBOT group. The HBOT group exhibited significantly greater hearing improvement (IPTW-adjusted difference: 7.6 dB, 95% CI 0.4–14.7; *p* = 0.038).

Both HBOT and ITCs remain viable options for salvage therapy in ISSNHL, with the choice depending on logistical considerations, including cost-effectiveness, patient accessibility, and local resources and expertise. Further studies with larger sample sizes and more rigorous designs are needed to refine the guidelines for salvage therapy in ISSNHL.

### 4.3. Other Therapies: Vasoactives and Rheologic Agents; Antivirals and N-acetylcysteine

Due to the multiple etiologies of sudden hearing loss, a wide variety of treatments are available. In this section, we discuss the main therapeutic alternatives currently in use, including drugs with vasodilatory effects, plasmapheresis, and supplements, such as N-acetylcysteine (Table 3).

#### 4.3.1. Vasoactives and Rheologic Agents

One possible cause of sudden hearing loss is vascular etiology, as previously mentioned, whether due to ischemic or thrombotic factors. Additionally, rheological and inflammatory alterations have also been studied in patients with ISSNHL, with recent studies reporting elevated plasma fibrinogen levels in patients with sudden hearing loss. From this vascular etiology, treatments for ISSNHL have emerged based on the vasodilatory action of drugs or agents (prostaglandin E1, calcium channel blockers, *Ginkgo biloba*, pentoxifylline, and defibrinogenation therapy), as well as apheresis therapy.

##### Prostaglandins

The mechanism of action of prostaglandin E1 (PGE1) consists of the dilation of peripheral blood vessels and an increase in peripheral cochlear blood flow [46]. Although PGE1 is used for some patients with ISSNHL, there is currently no evidence to recommend routine treatment with this medication.

A prospective double-blind RCT was conducted by Ogawa et al. [69]. After comparing combination treatment with steroids and PGE1 or a placebo for patients with ISSNHL, they found no significant differences regarding the overall hearing gain or rate of hearing improvement between the PGE1 and the placebo groups. However, hearing gains measured at 4 kHz or 8 kHz in the PGE1 group were found to be remarkably better than those in the placebo group. According to Okada et al. [70], in a multicenter database study of patients with severe ISSNHL, the group receiving combined PGE1 and steroids showed a substantially improved hearing prognosis. Additionally, this combination therapy was more effective in patients with the following characteristics: female, age ≥ 65 years old, latency to begin treatment < 4 days, and presence of vertigo.

In a retrospective study performed by Hara et al. [71] in 2020, the records of 67 patients with ISSNHL who were treated with systemic steroids and PGE1, with (*n* = 38) and without (*n* = 29) HBO additional treatment, were analyzed. PTA improvement (dB) was significantly greater in the HBO group [median hearing level (IQR): 38.0 (25.0, 47.0)] than that in the No-HBO group [median hearing level (IQR): 18.0 (5.50, 31.5)]. Multivariate logistic regression analysis revealed a significant positive correlation between pure tone audiometry improvement and hyperbaric oxygen therapy after adjustment for confounding factors (odds ratio = 7.42; 95% confidence interval = 2.37–23.3; *p* = 0.001).

##### Defibrinogenation Therapy

Defibrinogenation (DF) therapy using batroxobin has been used to treat ISSNHL. It is a compound extracted from snake venom and was originally used to treat occlusive arterial diseases.

A double-blind RCT carried out by Kubo et al. [72] showed that the overall recovery rate was remarkably higher in patients with ISSNHL receiving DF therapy than those with steroid therapy. In contrast, Jiang et al. [73], using a propensity score-matched approach, found no significant difference in the short-term hearing outcomes of ISSNHL patients receiving therapy with batroxobin or without it.

In another case–control retrospective study on 287 patients, Jiang and Zuo [74] found that DF can improve the efficacy of combination therapy for profound sudden sensorineural hearing loss exceeding 100 dB HL. Using batroxobin two to three times yielded the maximum overall improvement rate (41% vs. 16%).

In 2023, Weiss et al. [75] executed a double-blind, RCT, multicenter study with patients receiving an infusion of ancrod or a placebo (day 1) followed by subcutaneous administrations (days 2, 4, and 6). The study was terminated early due to slow recruiting (31 enrolled patients: 22 ancrod, 9 placebo). Both groups showed substantial improvement (ancrod: −14.3 dB ± 20.4 dB, placebo: −22.3 dB ± 13.7 dB), without any statistical differences being detected (*p* = 0.374). The placebo group revealed a complete response in 33.3% of the cases and a partial recovery in 85.7% of them. Plasma fibrinogen levels were reduced to a great extent by ancrod (baseline: 325.2 mg/dL, day 2: 107.2 mg/dL).

Recently, Wang et al. [76] published a retrospective study of 324 cases of total deafness due to ISSNHL with the aim of evaluating the efficacy of defibrinogen therapy and investigating the relationship between fibrinogen levels and auditory outcomes. Their results showed that patients in the experimental group, treated with batroxobin along with Ginaton and glucocorticoids, presented finer improvement in hearing compared to the control group. Additionally, they maintained low fibrinogen levels, which appears to be a positive prognostic factor.

##### Apheresis Therapy

Plasmapheresis (HELP or rheopheresis) involves filtering a patient’s blood through an extracorporeal device to remove lipids or specific pathogenic agents, such as LDL [77]. It is particularly indicated for patients with fibrinogen levels higher than 295 mg/mL [78]. The distinction between HELP apheresis and rheopheresis lies, among other factors, in the amount of heparin used, with the former utilizing a higher amount, leading to a greater reduction in LDL cholesterol, fibrinogen, and lipoprotein A.

A recent systematic review and meta-analysis conducted by Moreno-Herraiz et al. [78] found beneficial results regarding hearing recovery in patients with sudden hearing loss, particularly in those for whom corticosteroid treatment had been ineffective (57% remission rate). Their review included a total of 22 studies, 5 of which were randomized clinical trials. According to their findings, HELP apheresis showed a higher rate of hearing recovery compared to rheopheresis. However, the authors explained that the results may also be influenced by the effectiveness of plasmapheresis in reducing pro-inflammatory factors, such as cytokines or reactive oxygen species (ROS), thereby improving cellular stress conditions. Additionally, the significant heterogeneity in the analyzed studies must be considered.

#### 4.3.2. Antivirals

Although viral etiology has not been definitively proven as a cause of sudden hearing loss, some authors suggest that viral cochleitis could be related [79]. However, others propose that the patient’s immune status, rather than a viral infection, might determine the onset of hearing loss [80].

Currently, there is no evidence supporting the effectiveness of antiviral drugs in the treatment of sudden hearing loss [2]. Colin and Parnes, in 2007 [81], published a systematic review and meta-analysis on ISSNHL treatment, including four randomized clinical trials comparing corticosteroid treatment combined with antivirals to corticosteroid treatment with a placebo. They found no statistically significant differences between the two approaches.

Later, in 2017, Park SM et al. [82] also failed to demonstrate the efficacy of antiviral treatment for sudden hearing loss. Their study compared hearing outcomes in patients who received acyclovir in addition to corticosteroid treatment, but no statistically significant results were found.

#### 4.3.3. N-acetylcysteine

N-acetylcysteine (NAC) has several effects that are thought to be beneficial to cell stress in the inner ear. This medication can prevent hair cell apoptosis as a donor of reduced glutathione, fighting the damage generated by oxygenated radicals. Kranzer et al. [83] reported the otoprotective effect of NAC in preventing aminoglycoside-induced ototoxicity during tuberculosis treatment.

Angeli et al. [84], in their case–control study, were the first to report the beneficial effect of N-acetylcysteine (NAC) in ISSNHL. They examined patients treated with oral prednisone and intratympanic dexamethasone, either with or without NAC. At 6 months, the mean PTA improvements were 26.1 dB and 15.1 dB for the combination and single therapy groups, respectively (*p* = 0.046).

Since then, the use of NAC has gained popularity, with several studies supporting its use [83,84,85,86,87,88,89]. Notably, the study performed by Chen CH in 2017 [85], which compared the effect of NAC as a single therapy with oral corticosteroids, showed better hearing gains in patients treated with NAC.

A retrospective monocentric study carried out by Kouka et al. [89] investigated the effect of adding NAC to prednisolone treatment on 793 patients with ISSNHL. The study revealed that the important factors regarding a negative prognosis of hearing recovery were age > the median (OR 1.648; 95% confidence interval CI 1.139–2.385; *p* = 0.008), a diseased opposite ear (OR 3.049; CI 2.157–4.310; *p* < 0.001), pantonal ISSHL (OR 1.891; CI 1.309–2.732; *p* = 0.001), and the use of prednisolone alone without NAC treatment (OR 1.862; CI 1.200–2.887; *p* = 0.005). In conclusion, prednisolone treatment combined with NAC resulted in better hearing outcomes in patients with ISSHL than treatment without it.

**Table 3:** Main alternative treatment options. PT: pharmacological treatment, PGE1: prostaglandin E1, RCT: randomized controlled trial, HBOT: hyperbaric oxygen therapy, SR: systematic review, NAC: N-acetylcysteine, DF: defibrinogenation therapy.

**PROSTAGLANDINS**			
**Study**	**Main Finding**	**Type of Study**	**Level of Evidence**
Ogawa et al. [69]	Greater hearing gains at 4 or 8 kHz with PT + PGE1 treatment compared with PT alone	RCT	2
Okada et al. [70]	Better hearing prognosis with PT + PGE1 treatment than with PT alone	Retrospective multicenter database study	3
Hara et al. [71]	Significant improvement with PT + PGE1 + HBOT compared to PT + PGE1 alone	Retrospective study	3
**DEFIBRINOGENATION THERAPY**			
**Study**	**Main Finding**	**Type of Study**	**Level of Evidence**
Kubo et al. [72]	Overall recovery rate significantly higher with DF than PT	RCT	2
Jiang et al. [73]	No significant difference observed in short-term outcomes with or without batroxobin treatment	Retrospective study	3
Jiang and Zuo [74]	DF can improve efficacy of combination therapy for hearing loss > 100 dB HL	Retrospective case–control study	3
Weiss et al. [75]	No difference in hearing results with ancrod treatment vs. placebo	RCT	2
Wang et al. [80]	Significant improvement with DF + Ginaton + glucocorticoids treatment	Retrospective case–control study	3
**N-ACETYLCYSTEINE**			
**Study**	**Main Finding**	**Type of Study**	**Level of Evidence**
Kranzer et al. [81]	Otoprotective effect of NAC in preventing aminoglycoside-induced ototoxicity while tuberculosis treatment	SR/Meta-analysis	1
Angeli et al. [84]	Significant improvement with oral prednisolone + IT dexamethasone + NAC	Case–control	2
Chen et al. [85]	Better hearing improvement in patients treated with NAC alone	Case–control	2
Kouka et al. [89]	Better hearing outcomes with PT + NAC compared to PT alone	Retrospective observational study	3

## 5. Conclusions

This study reviewed numerous studies related to the treatment of SSHNL. The primary treatment for ISSNHL is usually based on oral steroids with or without intratympanic steroids. Nowadays, there seems to be less of a role for intravenous steroids.

Beyond steroids, the effectiveness of various treatment options remains unclear, but in certain cases, there are promising results. HBOT seems to play a role in severe–profound cases, as early as possible. When there is incomplete recovery after 7 days, rescue treatment must be started with intratympanic steroids with or without HBOT. There is an uncertain role for exploratory tympanotomy, but alternative treatments, such as prostaglandins or NAC, are promising. Therefore, due to the great diversity and controversy surrounding the latest therapeutic options, reviews that help us understand the treatment of SSNHL in a clear manner are necessary.

## Figures and Tables

**Table 1:** Methodology followed in this narrative review. Quality criteria.

**CLINICAL PRACTICE GUIDELINES/CONSENSUS STATEMENTS** Explicit scope and purpose. Systematic review of the literature.	**SYSTEMATIC REVIEW** Defined review protocol. Explicit and valid search strategy. Results of interest presented appropriately and coherently.
**CLINICAL TRIALS** Clear methodology with random assignment to treatment groups. Adequate sample size. Results clearly defined and relevant. Appropriate statistical analysis.	**META-ANALYSIS** Clear methodology. Comprehensive literature. Assessment of methodological quality of included studies. Appropriate statistical analysis. Heterogeneity assessment. Results presented clearly with effect measures and confidence intervals.

**Table 2:** Hyperbaric oxygen therapy. Main findings. Modified after Alter et al. (2023) [57]. RCT: randomized controlled trial; SR: systematic review; IT: intratympanic; PT: pharmacological treatment.

Hyperbaric Oxygen Therapy (HBOT)			
Study	Main Finding	Type of Study	Level of Evidence
Erygit et al. [60]	Significant improvement with HBOT.	SR/Meta-analysis	1
Lei et al. [61]	Advantage of both IT steroids + HBOT. Not notable variation in the effect between them when used as rescue treatment.	SR/Meta-analysis	1
Joshua et al. [53]	Significant improvement with the addition of HBOT (10.3 db).	SR/Meta-analysis	1
Tong et al. [62]	More significant improvement with HBOT + PT compared with PT alone; substantial improvement in patients with a younger age, rapidly started treatment, and mild–moderate hearing loss.	RCT	2
Cavaliere et al. [63]	The best results were obtained when monotherapy with HBOT was initiated in the first 7 days, while the HBOT and oral steroid combination showed more substantial benefits later.	RCT	2

## Data Availability

Data are contained within this article.
